# Chemical and Microscopic Characterization of the Yellow Passion Fruit Peel

**DOI:** 10.3390/molecules30214293

**Published:** 2025-11-05

**Authors:** Daniel Arrieta-Baez, Denise Larissa Díaz de la Torre, Héctor Francisco Mendoza-León, María de Jesús Perea-Flores, Mayra Beatriz Gómez-Patiño

**Affiliations:** 1 Instituto Politécnico Nacional—CNMN, Unidad Profesional Adolfo López Mateos, Col. Zacatenco, México City CDMX CP 07738, Mexico; darrieta@ipn.mx (D.A.-B.); hfmendoza@ipn.mx (H.F.M.-L.); mpereaf@ipn.mx (M.d.J.P.-F.); 2 Instituto Politécnico Nacional—ENCB, Unidad Profesional Adolfo López Mateos, Col. Zacatenco, México City CDMX CP 07738, Mexico; deniselarissad@gmail.com

**Keywords:** yellow passion fruit, cutin, CPMAS ^13^C NMR, UHPLC-MS, CLSM, SEM

## Abstract

Passion fruit (*Passiflora edulis* f. *flavicarpa*), commonly known as yellow passion fruit, is widely grown across tropical and subtropical regions worldwide, with Brazil as one of the top producers. Mexico also produces a significant amount of this variety, mainly for juices, jams, or flavoring in desserts. Since this fruit is highly perishable with a short shelf life, it needs to be consumed or used quickly. Although different preservation methods have been suggested, no structural analyses of the peel have been performed to improve these processes. This study aimed to analyze the structural and chemical properties of the peel’s cuticular matrix to better understand water loss. CPMAS ^13^C NMR analysis revealed a matrix containing polysaccharides, a small amount of aliphatics, and a notable group of aromatic signals that may indicate lignin presence. This was supported by alkaline hydrolysis, which achieved only 30% hydrolysis. Soluble compounds identified included hexoses, palmitic acid, stearic acid, and derivatives of ferulic and caffeic acids, the latter being parts of lignin monomers. MCL and SEM analyses showed features similar to cutans, including pores along the structures. The BET surface area measurement indicated that the insoluble cuticular material (ICM) has a significant specific surface area. The lignin in the yellow passion fruit peel gives the shell toughness, which, along with its pores, may contribute to dehydration and a short shelf life.

## 1. Introduction

The genus *Passiflora* consists of more than 500 species, distributed in tropical and subtropical regions. One of the most cultivated and consumed species is *Passiflora edulis* f. *flavicarpa*, also known as yellow passion fruit [1,2]. This fruit has a thick, hard shell of a vibrant yellow color with brown seeds. It has an acidic pulp and a characteristic aroma derived from various volatile compounds, which makes it ideal for the food industry, where it is mainly used to prepare juices and jams [3,4,5]. The general composition of passion fruit is: peel 50%, pulp 30 to 40% (which is the most important), and seed 10 to 15%. These species have also been historically used in traditional medicine as a diuretic, digestive aid, sedative, and antidiarrheal, as well as in the treatment of cough, constipation, insomnia, infant colic, joint pain, and more recently, as sedatives and anxiolytics [2,6,7]. Currently, its demand is increasing, and it is cultivated in many countries, with Brazil being one of the world’s largest producers and consumers [8]. In Mexico, the cultivation of passion fruit has become increasingly important, with the state of Veracruz leading the way as the primary producer, cultivating 531 hectares [9]. Its primary consumption is in the form of juices due to its low shelf life. In fact, the marketing of passion fruit for fresh consumption is reduced due to rapid changes in its appearance, affecting commercial losses [10,11,12,13]. It has been demonstrated that shelf life is influenced by moisture loss, resulting from intense shriveling, which can lead to a 21% weight loss in 21 days at 18 °C and 80% relative humidity [5]. A series of processes have been proposed in various studies to increase shelf life, including modified and controlled atmospheres under refrigeration at 4 °C, modified atmosphere packaging using biopolymer coatings and films, chemical substances (such as 1-MCP), and nanoparticles [10,14,15,16]. However, it remains unclear what process in the shell causes this rapid loss of moisture, and how to make the proposed methods more effective. For the industrial sector, understanding the shelf life of products is crucial, particularly for adapting storage conditions and time.

Fruits and vegetables contain a biopolyester formed by a polymeric network of fatty acids, typically C16 and C18 aliphatic (cutin), polysaccharides, and intracuticular and extracuticular waxes, collectively referred to as the cuticle. This cuticle serves as a barrier protecting fruits and leaves from mechanical damage and pathogen attacks, while also offering protection from UV rays. Most importantly, it allows for nutrient exchange and prevents fruit from becoming dehydrated. Today, it is known that the cuticle plays a significant role not only during fruit development but also during ripening and the post-harvest period [17]. In recent years, the scientific community has become increasingly interested in the cuticle, as changes that occur during harvest and shelf life can lead to economic losses and the high production of agro-industrial waste derived from post-harvest losses. For this reason, it is essential to continue the chemical and structural characterization of the cuticles and cutins of fruits that are widely consumed internationally. Until now, there have been few studies of the yellow passion fruit peel, which is mainly composed of three different tissues: a hard, waxy, deep yellow epicarp (the external epidermis); a white and spongy parenchyma corresponding to mesocarp, and the endocarp [5]. Most of these studies are related to the presence of polyphenol compounds and pectin [18,19]. In fact, *P. edulis* f. *flavicarpa* pericarp, obtained from agro-industrial residues of yellow passion fruit (by-products of its processing for obtaining pulp or juice), has been used as a good raw material for pectin production under different conditions of extraction with a satisfactory quality, as demonstrated in studies of jelly production [18,20]. In this regard, these works were focused on optimizing pectin extraction from peel residues due to its positive role in health, including serving as an antioxidant, protecting the gut biota, and being hypolipidemic [21]. Beyond these works, there are no studies that chemically and microscopically indicate how the passion fruit peel is structurally constituted.

This work aimed to study the structural and chemical morphology of the cuticular matrix of yellow passion fruit peel to better understand the water loss process. Based on this, the yellow passion fruit peel was analyzed using reported enzymatic methods to separate the polysaccharides present in the mesocarp. Under the CPMAS ^13^C NMR analysis, the insoluble cuticular material (ICM) is mainly composed of a polysaccharide domain (cellulose) and lignin. This was corroborated by alkaline hydrolysis, which revealed that the main components of these biopolymers are present. Confocal and Scanning Electron Microscopy (CLSM and SEM) revealed atypical cuticular structures compared to other fruit cutins, characterized by a peculiar presence of pores. BET surface area assessment showed that the insoluble cuticular material possessed a significant specific surface area. These observations could lead to significant improvements, increasing the shelf life of the fruit and reducing agro-industrial waste, ultimately resulting in lower economic losses.

## 2. Results and Discussion

As is well known, the yellow passion fruit peel is made up of a large amount of pectin; therefore, to analyze the chemical and structural components, enzymatic hydrolysis was carried out, according to protocols previously reported to analyze the peels of other fruits [22,23], and solvent extractions were performed to study the cuticular material present in the peel.

### 2.1. Ultra-High-Performance Liquid Chromatography–Mass Spectrometry Electrospray (UHPLC-ESI) Analysis of Fractions 1 and 2

**Figure 1:**
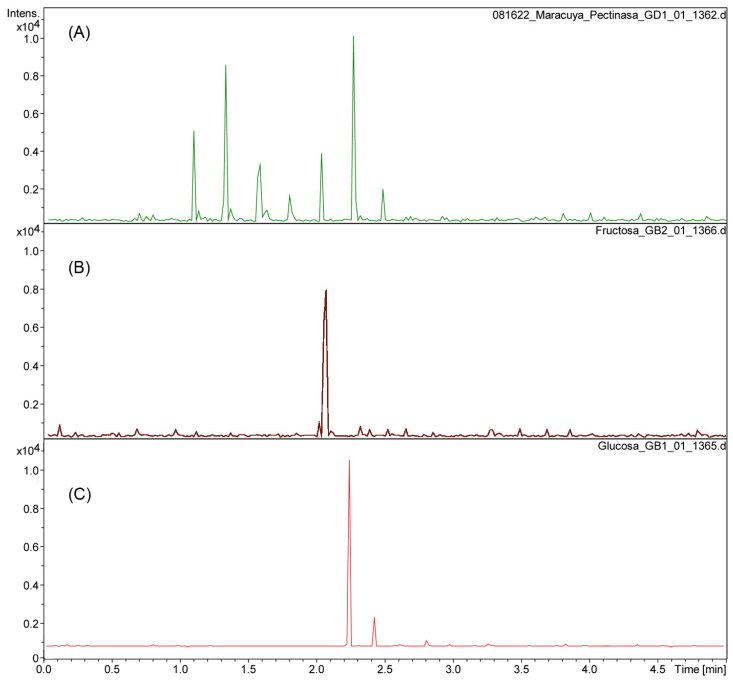
Chromatogram of pectinase reaction from *P. edulis* f. *flavicarpa* (**A**) using fructose (**B**) and glucose (**C**) as references.

The aqueous residue fractions 1 and 2 from the enzymatic reactions to isolate the cuticular material of *P. edulis* f. *flavicarpa* were analyzed using UHPLC-ESI(-) to determine the monosaccharide composition present. The enzymatic reaction, particularly with pectinase, reveals the presence of glucose (rt 2.3), fructose (rt 2.1), galacturonic acid (rt at 1.1, 1.3, 1.6 and 1.8 min; *m*/*z* 193.03), and other sugars, including rhamnose, arabinose, and galactose (Figure 1A,B).

These observations are essential since the yellow passion fruit peel has a significant amount of pectin. Pectin has been described as a hetero-polysaccharide located in the middle lamella of the plant cell wall that acts as “biological glue” to bind adjacent cells together, providing tissue strength and integrity. Pectin has been utilized for gelification, emulsion stabilization, and the delivery of nutritional fiber [17]. Recently, research has focused on pectin extraction from various fruits, including those of the genus *Passiflora*, for applications in the food and pharmaceutical industries. This effort utilizes both conventional and unconventional extraction methodologies to promote the creation of high-value, sustainable products [1].

Enzymatic hydrolysis was then carried out using cellulase and hemicellulase (Fraction 2) for the remaining polysaccharides from the yellow passion fruit peel. This reaction shows only the presence of glucose, as expected.

Once the enzymatic reactions were completed, the insoluble cuticular material was stirred in a CHCl_3_:MeOH (1:1, *v*/*v*) mixture to complete the extraction of the soluble compounds. After this, a yield of 43.6% of ICM was recovered.

### 2.2. Solid State NMR (CPMAS ^13^C NMR) Analysis of the ICM of Passiflora edulis f. flavicarpa

**Figure 2:**
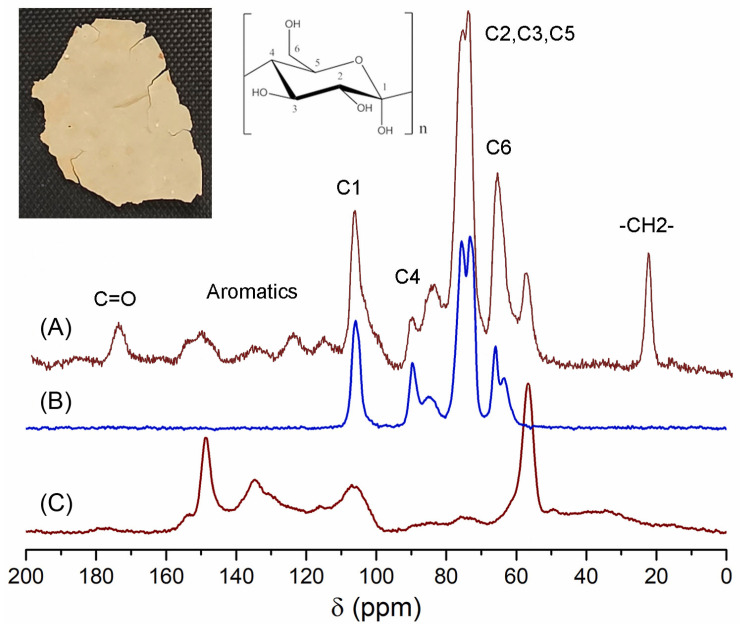
CPMAS ^13^C NMR spectra of (**A**) ICM of the *P. edulis* f. *flavicarpa*, (**B**) cellulose and (**C**) lignin.

The ICM of *P. edulis* f. *flavicarpa*, previously obtained from enzymatic reactions, was analyzed by CPMAS ^13^C NMR. The main resonance assignments were as follows: bulk methylenes at 20 ppm, an important proportion of aromatics and olefins (110–150 ppm), and carbonyl groups (173 ppm). Main peaks were assigned to carbohydrate moieties (C6 at 60 ppm, C2–C5 at 70–75 ppm, C4 at 83 ppm, and C1 at 105 ppm) (Figure 2A). These signals could be assigned to the cellulose (Figure 2B) that remains in this important biopolymer. Another important observation was the presence of a group of aromatic signals that could be associated with a lignin domain. To confirm the presence of lignin, a reference was used, and, according to Figure 2C, peaks at 59, 135, and 150 ppm correlate with those present in *P. edulis* f. *flavicarpa* ICM. Under this analysis a presence of ≈29.2% of lignin was calculated.

Most of the signals observed have been previously reported for other cutins. However, there are notable differences in some signals.

**Figure 3:**
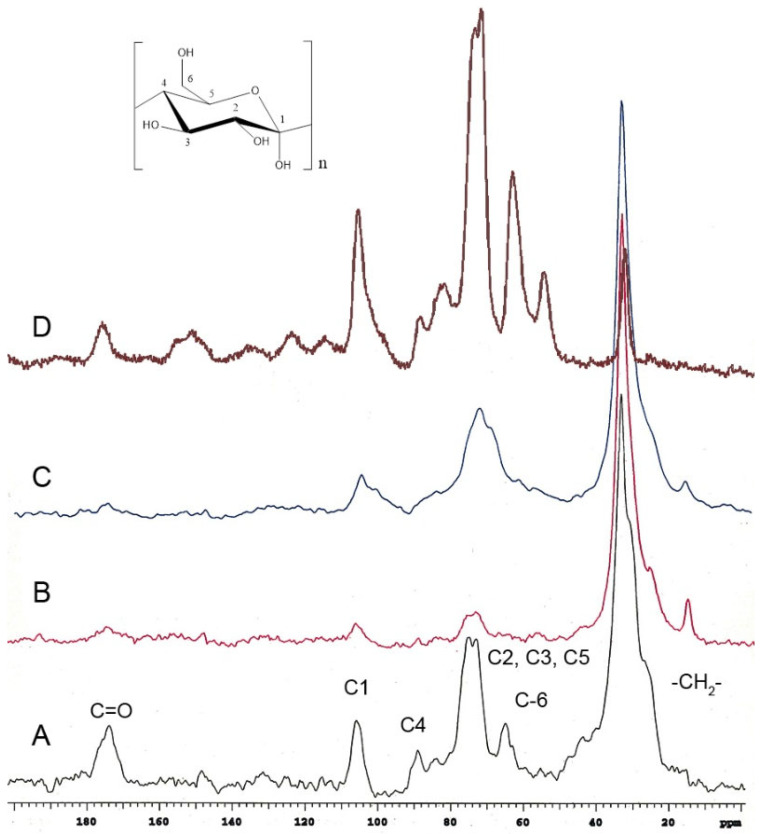
CPMAS ^13^C NMR spectra of cutins previously analyzed in (**A**) red tomato, (**B**) grapefruit, (**C**) lime, compared with the (**D**) ICM of *P. edulis* f. *flavicarpa* ICM.

Red tomato (Figure 3A) [24], grapefruit (Figure 3B) [25], and lime (Figure 3C) [25] showed that the important domain belongs to the aliphatic compounds (20–40 ppm), and these peaks in *P. edulis* f. *flavicarpa* ICM are of very low intensity (Figure 2A and Figure 3D). The other notable difference was the presence of aromatic signals, which are absent in other cutins. The presence of aromatic signals in other cutins has been related to phenolic compounds, which usually achieve the function of protection from the sun’s rays, but to date, there is no association with the presence of other cuticular matrices present in plants, such as lignin or suberin, non-saponifiable and non-extractable biopolymers. Usually, these non-extractable cuticular matrices are present in the roots and stems of plants. However, they have been reported in the leaves of *Agave*, where they are commonly referred to as cutan. So, cutin is defined as a depolymerized and solubilized biopolymer upon saponification, while cutan is the non-saponifiable and non-extractable polymer found in certain cuticles. Based on these observations, the presence of a non-extractable biopolymer such as lignin (Figure 2C), which could be present in the cuticular matrix of *P. edulis* f. *flavicarpa*, is not typically found in fruit peels. This is the first report where this type of cuticular matrix is described in fruits, and it is an important observation, as the yellow passion fruit peel exhibits a different hardness compared to other fruit peels. Usually, the softness and flexibility observed in other cutins have been related to the presence of aliphatic components and polysaccharides [26]. The mechanical properties of the cuticles are related to the monomeric aliphatic composition and the interaction with hydroxyl groups of the polysaccharides present to increase their hydrophilic character, resulting in greater elasticity [27]. The presence of aliphatics is very low in the cuticular matrix of *P. edulis* f. *flavicarpa*, and it is well known that lignin shows a very rigid structure due to the aromatic network. This important finding may contribute to explaining the very short shelf life of yellow passion fruit.

### 2.3. Confocal Laser Scanning Microscopy Analysis of the ICM of Passiflora edulis f. flavicarpa

CLSM has become a valuable tool for analyzing the autofluorescence of fruit cutins, as treatments or physical sectioning of the samples are not required, thereby leaving the cutins unaltered. ICM from *P. edulis* f. *flavicarpa* was analyzed without artificial staining or physical sectioning, allowing for the observation of its structural organization due to autofluorescence. Depth-coded 3D images were obtained from both the inner and outer surfaces of the ICM of *P. edulis* f. *flavicarpa.*

**Figure 4:**
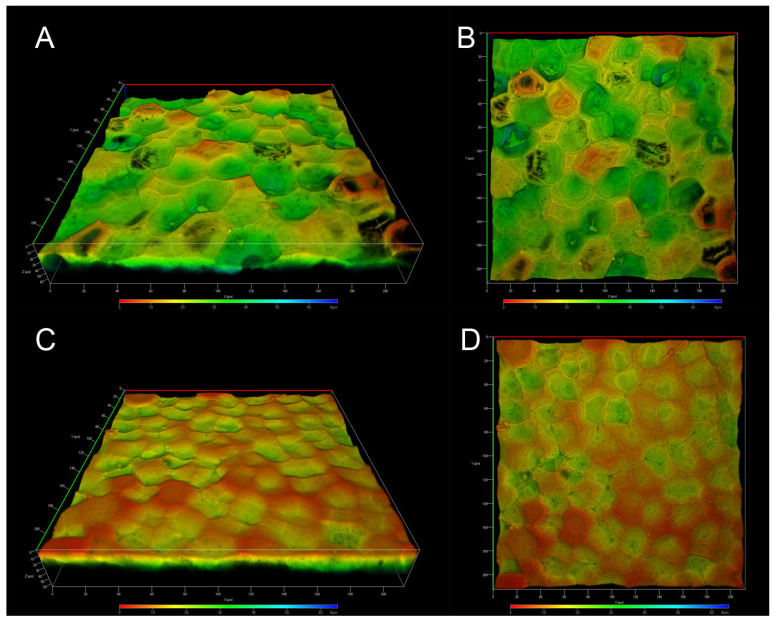
CLSM micrographs of the ICM from *P. edulis* f. *flavicarpa*: inner face (**A**,**B**), and outer face (**C**,**D**).

Images of the inner face clearly show the presence of concavities across the entire surface, depicted as the deeper zones in green; shallow zones are also visible in orange, although the deeper ones are more common (Figure 4A,B). In the images of the outer face, we can also observe the concavities, but their depth is shallower and there are fewer of them, allowing us to observe more superficial areas (Figure 4C,D). According to solid-state NMR analysis, differences in the ICM of the yellow passion fruit compared to other fruit peels were expected. In fact, one of the main differences is the absence of anticlinal walls, which are characteristic of the tomato, lemon, grapefruit, and other cutins. These anticlinal walls have been reported to be formed from long-chain aliphatic acids and polysaccharides [23]; therefore, the absence of aliphatic compounds in the ICM of *P. edulis* f. *flavicarpa* affects the formation of these structures. On the other hand, the concavities observed in Figure 4 are more closely related to those observed in *Agave* leaves, where the presence of cutin and cutan has been reported. So, this morphological conformation could be linked to the chemical composition, specifically the presence of lignin, in the ICM of yellow passion fruit.

Another important observation is the presence of small pores throughout the sample (Figure 4A,B) that make the surface structure of this ICM of *P. edulis* f. *flavicarpa* entirely different from other surface cutins reported. In the green tomato cutin, some pores have been reported [26], but they are related to the connection with the calyx of the fruit. So, this is the first time that these concavities and small pores in the cuticular area of the peel fruit have been observed.

These micrographs bring us closer to a unique description and elucidation of the structures of this particular cuticular material, which, at first glance, is entirely different from the previously analyzed cutins. To gather more details about its morphological structure, SEM analysis was used.

### 2.4. Scanning Electron Microscopy Analysis of ICM of Passiflora edulis f. flavicarpa

SEM was also used to analyze the samples examined by CLSM for better traceability. Micrographs were taken from the inner and outer faces of the cuticular material of yellow passion fruit, both uncoated and coated, to show details of some structures.

**Figure 5:**
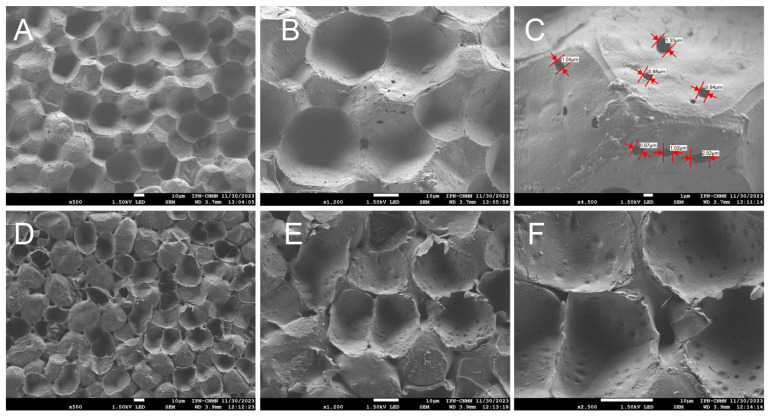
SEM micrographs of the ICM from *P. edulis* f. *flavicarpa*: inner face (**A**–**C**), and outer face (**D**–**F**).

Images of the inner face of the ICM of the yellow passion fruit reveal unique structures not previously observed in any of the cutins studied before. Concavities and protuberances are seen, scattered randomly, creating a surface that appears heterogeneous (Figure 5A,B). This contrasts with cutins such as those of the Mexican husk tomato [26], where irregular geometric shapes are observed, bordered by ribs and positioned in the same focal plane. There are no spaces between these geometric structures that form the cutin, the ribs being the shapes that delimit them, which makes this cuticular material a highly resistant and malleable matrix.

As mentioned previously, the ICM of yellow passion fruit is composed mainly of cellulose and aromatic compounds, unlike other cutins such as lemon and grapefruit, where aliphatic compounds predominate, followed by polysaccharides [25]. It is believed that they may be related to the support of the cutin structure, influencing greater elasticity and lower porosity, which in turn contribute to the peel’s resistance to mechanical damage and reduced dehydration, resulting in a longer shelf life [28]. In Figure 5C open pores are also observed throughout the entire area, with a circumference ranging from 0.88 μm to 2.02 μm, another unique feature of this ICM. These open spaces support the idea that, since the composition is not primarily aliphatic, forming a close network with the polysaccharides, pores are formed, which could influence greater water exchange with the environment and cause substantial moisture loss during the fruit’s shelf life.

**Figure 6:**
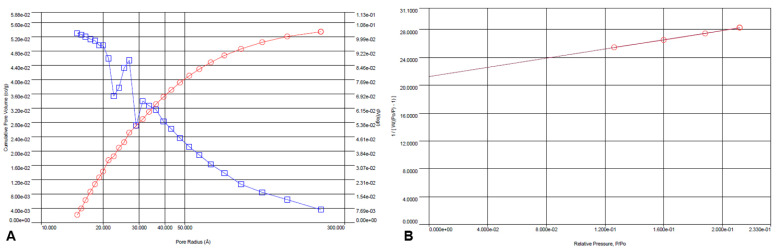
BJH-adsorption pore size distribution curve (v, red circles; dV(logr), blue squares) (**A**) and multi-point BET analysis of the ICM (**B**) from *P. edulis* f. *flavicarpa*.

Using the BJH method and nitrogen adsorption/desorption BET, the surface parameters of the ICM were determined. These analyses showed that the ICM had a significant specific surface area of 64.199 m^2^/g, a pore volume of 0.053 cm^3^/g, and an average pore diameter of 1.5 nm (Figure 6 A,B).

On the outer face of the ICM, concavities and protuberances can also be seen, with evident spaces between one structure and another, supporting the idea that this ICM is highly porous and susceptible to dehydration. These concavities exhibit an elongated form, distinct from those on the inner face. A significant number of pores can also be seen throughout the entire area (Figure 5D–F). This type of structural organization, characterized by the arrangement of cell traces, has been reported only in *Agave sisalana* tissue [29].

The authors report that, among other factors, the deposition of lignin, even in minimal amounts, provides the plant with mechanical support and strength, and contributes along with cellulose to the structural organization of this plant tissue.

The coated images of the inner and outer faces of the ICM revealed the presence of some unknown artifacts, never seen before in other cutins, which is why an elemental analysis was performed.

**Figure 7:**
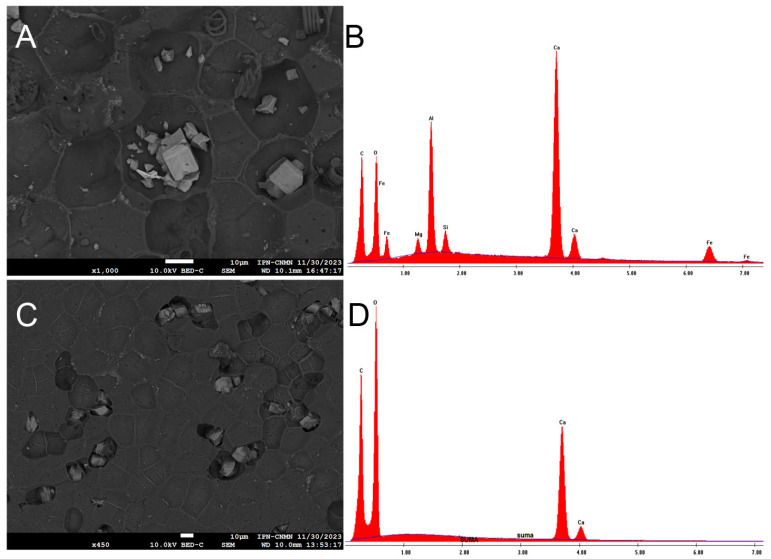
SEM micrograph of the deposits of metals in the inner face of *P. edulis* f. *flavicarpa*, and its elemental analysis (**A**,**B**) and SEM micrograph of the deposits of calcium in the outer face of *P. edulis* f. *flavicarpa*, and its elemental analysis (**C**,**D**).

In the coated images of the inner face of the ICM, deposits of metals such as silicon, aluminum, and iron can be observed, as well as elements like calcium and magnesium, and some aliphatic components (spiral-shaped), all of which are situated in the concavities (Figure 7A,B). Although it is known that yellow passion fruit does not absorb metals, the idea that it could become an option for soil bioremediation cannot be ruled out due to this evidence. On the other hand, it would be worth analyzing the juice of this fruit to discard the idea that metals are also accumulating in the pulp. Shanmugama et al. (2020) [30] reported a high scavenging metal chelation activity in the pulp of *Passiflora leschenaultii*, a behavior also observed in other *Passiflora* species.

In the coated images of the outer face of the ICM, calcium deposits are visible in the existing concavities, and pores are also evident throughout the area (Figure 7C,D).

### 2.5. Direct Injection Electrospray Mass Spectrometry (DI-ESI-MS) Analysis of the KOH Hydrolyzed of ICM of Passiflora edulis f. flavicarpa

To analyze the main chemical components in the ICM of *P. edulis* f. *flavicarpa*, an alkaline hydrolysis was performed according to protocols established for other cutin fruit studies [23,26,28]. After 24 h, soluble products were obtained, and the yield of the hydrolysis ranged from 25 to 30%. The yield was very low compared to that previously reported for other cuticles [24,25,26,28]. This difference in reaction yield could be attributed to the presence of lignin, a non-saponifiable polymer, in the cuticular matrix of *P. edulis* f. *flavicarpa*. Reactions used to depolymerize lignin involve more complex reactions such as alkaline hydrolysis, followed by transesterification, and hydrogenolysis with LiAlH_4_ in THF [31]. So, the reaction used was less effective in carrying out the depolymerization.

**Figure 8:**
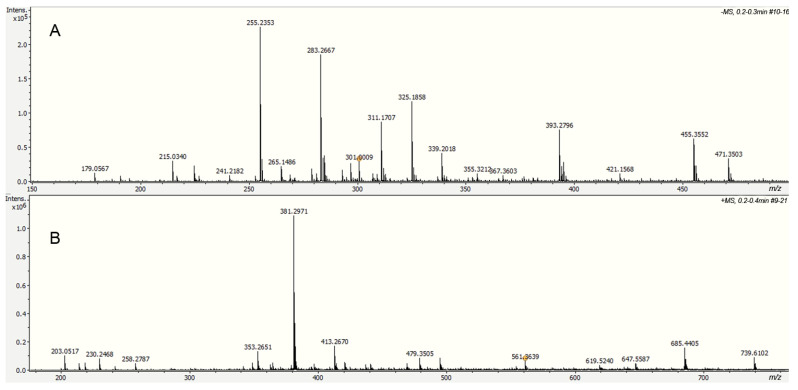
Spectra of the soluble fraction from the alkaline hydrolysis of the ICM of *P. edulis* f. *flavicarpa* using DI-ESI (−) (**A**) and DI-ESI (+) (**B**).

Alkaline hydrolysis products were analyzed directly by electrospray ionization in negative mode (DI-ESI-) (Figure 8), revealing a different molecular weight distribution compared to that observed in other cutins. Ten compounds were identified based on their exact molecular weight and MS/MS analysis; their relative concentrations are shown in Table 1. Palmitic and stearic acids were identified in the aliphatic group, which are usually present as cuticular waxes. However, neither 10,16-dihydroxyhexadecanoic acid nor other long-chain aliphatic acids, main components of cutins, were identified in the hydrolyzed fraction. Depolymerization studies in cuticles containing cutin and cutan, such as *Agave americana*, specifically hydrolyze the cutin domain, yielding primarily C18 fatty acid-derived monomers, including 9,10,18-trihydroxyoctadecanoic acid, 9,10-epoxy-18-hydroxyoctadecanoic acid, and 10,16-dihydroxyhexadecanoic acid. However, the cutan part remains, and its structure is only partially understood [32]. The absence of long-chain aliphatic acids in the ICM of *P. edulis* f. *flavicarpa* suggests that cutin is not present, and the aromatic part observed in CPMAS ^13^C NMR corresponds only to the lignin derivative fractions. Besides these compounds, a small amount of hexoses (glucose and fructose, *m*/*z* 179.0567) and some phenolic compounds were detected under this analysis.

Lignin is a heterogeneous biopolymer derived especially from three monolignols (p-coumaryl, coniferyl, and sinapyl alcohols). At the same time, suberin is characterized by the presence of two domains: a poly(aliphatic), typically derived from long-chain aliphatic acids, and a poly(phenolic) domain derived from monolignol and sterified hydroxycinnamic acids (ferulic, caffeic, cinnamic, and cumaric acids). From the analysis of the same fraction in positive mode (DI-ESI+), the presence of caffeic and ferulic acid derivatives (Table 2) was observed, indicating that these compounds could correspond to the presence of lignin in the cuticular matrix of *P. edulis* f. *flavicarpa* ICM, according to the CPMAS ^13^C NMR analysis. However, more studies are necessary to understand the nature of this untractable part of the cuticular matrix.

This peel of the yellow passion fruit is notable because it lacks cutin and contains only a lignin domain in the cuticular structure, whereas most fruit cuticles are characterized by the presence of cutin (poly (aliphatic domain)) and polysaccharides.

## 3. Materials and Methods

### 3.1. Isolation of Passiflora edulis f. flavicarpa ICM

Yellow passion fruits were purchased at a local market in Cuetzalan, Puebla, México (20°02′00″ N 97°30′00″ W/20.033333333333, −97.5). The fruit was ripe and in optimal condition for consumption, with a uniform yellow color and no bruises. The entire peel of each fruit was used to isolate the ICM. They were peeled by hand, without tools, and the pulp was removed. ICM were obtained using the methodology previously described by Arrieta-Baez et al. (2024) [26]. Briefly, peels were treated with a solution of 50 mM sodium acetate (Sigma Aldrich, Toluca, Mexico), pH 4, containing *Aspergillus niger* pectinase (Sigma Aldrich, Toluca, Mexico) (10 mL/L, equivalent to 2500 units of pectinase) and stirred for three days at 150 rpm and 44 °C. Afterward, the cuticle material was washed with tap water (Fraction 1) and treated with a solution of sodium acetate 50 mM, pH 5, containing *A. niger* cellulase (Sigma Aldrich, Toluca, Mexico) (1 g/L, equivalent to 1200 units of cellulase) and hemicellulase (Sigma Aldrich, Toluca, Mexico) (1 g/L, equivalent to 1500 units of hemicellulose), stirring for three days at 150 rpm at 37 °C. Finally, the ICM was washed with tap water (Fraction 2), filtered, and subjected to another wash with a 1:1 (*v*/*v*) mixture of methylene chloride and methanol for 2 h to remove residual ICM compounds such as monosaccharides, soluble polysaccharides, and waxes. Fractions 1 and 2 were analyzed by Ultra-High-Performance Liquid Chromatography–Mass Spectrometry (UHPLC-MS) (Billerica, MA, USA). The ICM was also analyzed by Cross Polarization Magic Angle Spinning CPMAS ^13^C NMR (Billerica, MA, USA), Confocal Laser Scanning Microscopy (CLSM) (Jena, Germany), Scanning Electron Microscopy (SEM) (Almere, The Netherlands), and the Brunauer–Emmett–Teller (BET) (Norcross, GA, USA) method.

### 3.2. Ultra-High-Performance Liquid Chromatography–Mass Spectrometry (UHPLC-MS) Analysis of Fractions 1 and 2

An Ultimate 3000 ultra-performance liquid chromatography (UHPLC) system (Dionex Corp., Billerica, MA, USA) with photo diode array detection (PAD) was coupled to a Bruker MicrOTOF-QII system by an Electrospray Ionization (DI-ESI) interface (Bruker Daltonics, Billerica, MA, USA) for chromatographic and Mass Spectrometry (MS) analysis. For chromatographic separation, a Hypersil C18 column (3.0 μm, 125 × 4.0 mm) (Sigma Aldrich, Toluca, Mexico) was used. The mobile phase consisted of 0.1% formic acid in water (A) and acetonitrile (B) using an isocratic program of 5–95% (B) in 5 min. The solvent flow rate was 0.5 mL/min, the column temperature was set to 30 °C, and the detection wavelength was 254 nm. The conditions of MS analysis in the negative ion mode were as follows: drying gas (nitrogen), flow rate, 8 L/min; gas temperature, 180 °C; scan range, 50–3000 *m*/*z*; end plate offset voltage, −500 V; capillary voltage, 4500 V; nebulizer pressure, 2.5 bar.

The accurate mass data of the molecular ions were processed through the software DataAnalysis 4.1 (Bruker Daltonics Technical Note 008, 2004). The widely accepted accuracy threshold for confirmation of elemental compositions was established at 5 ppm.

### 3.3. Solid State NMR Spectroscopy Analysis of Passiflora edulis f. flavicarpa ICM

ICM was analyzed using standard CPMAS ^13^C NMR experiments performed on a Bruker AVANCE III 400 MHz wide-bore spectrometer (Billerica, MA, USA) equipped for solid-state NMR. The resonance frequency was 100.61 MHz, with a customary acquisition time of 60.8 ms, a delay time of 2 s between successive acquisitions, and a CP contact time of 1.5 ms. Typically, each 30 mg sample was packed into a 5 mm Double Resonance (X/H-F) WB Static Solid-State Probe (Billerica, MA, USA) and then spun at 11 kHz at room temperature for approximately 8 h. No spinning sidebands were observed upon downfield from the major carbonyl, aromatic, or aliphatic carbon peaks, presumably due to motional averaging and/or excessive broadening of such features.

### 3.4. Confocal Laser Scanning Microscopy Analysis of Passiflora edulis f. flavicarpa ICM

For CLSM analysis, each sample was mounted on glass slices and observed using a CLSM (LSM 710 NLO, Carl Zeiss, Jena, Germany) with an objective of EC Plan-Neofluar 10×/0.3. The laser wavelength excitation was 405, 488, 561, and 633 nm, all simultaneously. The capture mode employed was a spectral imaging technique that automatically outputs separate channels for multiple labeled samples. This tool detects the autofluorescence signal of cuticular material. The z-stack images (3D images) were captured using the software ZEN 2010 (Carl Zeiss, Germany), at a resolution of 512 × 512 pixels in RGB color, and stored in TIFF format at 8 bits.

### 3.5. Scanning Electron Microscopy Analysis of Passiflora edulis f. flavicarpa ICM

ICM of yellow passion fruit were mounted on aluminum holders using a conductive double-sided adhesive tape (Plannet Plano, PA, USA), 1 min coated with carbon (60:40) at 25 mA using an SPI sputter coater and examined in a field emission scanning electron microscope (JEOL JSM-7800F, Almere, The Netherlands) at 1.5 kV. The sputter conditions, depositing a carbon coat with a thickness of approximately 10 nm, were optimized for the acceleration voltage in the scanning electron microscope. The samples were observed in secondary electron mode and backscattered electron mode.

### 3.6. Brunauer–Emmett–Teller (BET) Analysis of Passiflora edulis f. flavicarpa ICM

Porosity and surface area were evaluated with a Quantachrome NOVA 4200e (Norcross, GA, USA) Surface Area and Pore Size Analyzer. Prior to analysis, the samples were degassed at 100 °C for 12 h under vacuum to remove moisture and gases.

### 3.7. Alkaline Hydrolysis Passiflora edulis f. flavicarpa ICM

500 mg of ICM was hydrolyzed with 1 M methanolic KOH, this mixture was stirred at room temperature for 5 days. ICM monomers were extracted with CHCI_3_-MeOH using standard procedures [24]. The dried extract was dissolved in 1.0 mL of CHCI_3_-MeOH (17:3) and filtered through glass wool into clear glass vials. Finally, the extract was analyzed by DI-ESI-MS in positive and negative ion mode.

### 3.8. Direct Injection Electrospray Mass Spectrometry (DI-ESI-MS) Analysis of the KOH Hydrolyzed of Passiflora edulis f. flavicarpa ICM

DI-ESI-MS analysis was performed on a Bruker MicrOTOF-QII system, utilizing an electrospray ionization (DI-ESI) interface (Bruker Daltonics, Billerica, MA, USA) operated in both positive and negative ion modes. A solution of 10 µL of the sample, resuspended in 1 mL of methanol, was filtered through a 0.25 µm polytetrafluoroethylene (PTFE) filter and diluted 1:100 with methanol. Diluted samples were directly infused into the DI-ESI source and analyzed in positive and negative ion mode. Nitrogen was used with a flow rate of 4 L/min (0.4 Bar) as a drying and nebulizer gas, with a gas temperature of 180 °C and a capillary voltage set to −4500 V. The spectrometer was calibrated with a DI-ESI-TOF tuning mix calibrant (Sigma-Aldrich, Toluca, Estado de México, Mexico).

## 4. Conclusions

The insoluble cuticular material of the peel of yellow passion fruit (*Passiflora edulis* f. *flavicarpa*) was isolated and analyzed. This biopolyester primarily exhibited cellulose and aromatic signals that could be attributed to lignin, as indicated by the CPMAS ^13^C NMR, with a minimal presence of aliphatic compounds. This atypical biopolymer may be more closely related to cutan, which is present in certain plants, such as the agave (*A. americana*). The low yield obtained from the alkaline hydrolysis, where only palmitic and stearic acids were identified, corroborates this observation. In fact, none of the long-chain aliphatic acids, such as 10,16-dihydroxyhexadecanoic acid, the main component of the fruit cutins, were identified in the hydrolyzed fraction. This is the first time that a non-saponifiable and non-extractable cutan-like polymer is reported in a fruit peel. The presence of lignin gives the polymeric matrix a different hardness than that of other fruit peels and may be responsible for pore formation throughout the entire surface, a characteristic also reported for agave cutan. The presence of these pores gives yellow passion peel a distinct characteristic, making it more prone to dehydration and shortening its shelf life. Furthermore, the discovery of metals deposited in the concavities of the ICM opens a new avenue for the study of this fruit as a metal binder. This also raises the question of whether these metals are being consumed in the pulp and juice, so further studies are needed.

## Figures and Tables

**Table 1:** DI-ESI (−) Identification of the main compounds in the soluble fraction of the KOH.

Compound	Formula	[M − H]^−^ W_exact_	[M − H]^−^ MW_detec_	Error ^a^	%Rel ^b^
Hexose	C_6_H_12_O_6_	179.0561	179.0567	−3.4	1.1
(E)-3-(4-oxo-4H-chromen-3-yl) acrylic acid	C_12_H_8_O_4_	215.0350	215.0337	4.5	2.5
Palmitic acid	C_16_H_32_O_2_	255.2330	255.2352	5.2	21.6
Stearic acid	C_18_H_36_O_2_	283.2643	283.2668	3.1	19.6
4,9-Dioxopyrano[2,3-g]chromene-2,7-dicarboxylic acid	C_14_H_6_O_8_	300.9990	301.0008	3.9	0.4
Acitretin	C_21_H_26_O_3_	325.1809	325.1862	1.5	15.1
2-[(E)-5-(4-methoxy-2,3,6-trimethylphenyl)- 3-methylpent-2-enyl] benzene-1,4-diol	C_22_H_28_O_3_	339.1966	339.2008	2.4	5.6
Galloxanthin	C_27_H_38_O_2_	393.2858	393.2790	0.8	13.4
Oleanolic Acid	C_30_H_48_O_3_	455.3531	455.3550	1.3	11.8
Maslinic Acid	C_30_H_48_O_4_	471.9480	471.3509	3.5	6.80

^a^ Error [ppm]: Absolute value of the deviation between measured mass and theoretical mass of the selected peak in [ppm]. ^b^ Percentage of compound detected in the DI-ESI analysis. Results correspond to the products yield in triplicate.

**Table 2:** DI-ESI (+) Identification of the main compounds in the soluble fraction of the KOH.

Compound	Formula	[M − H]^+^ MW_exact_	[M − H]^+^ MW_detec_	Error ^a^	%Rel ^b^
Hexose (Na adduct)	C_6_H_12_O_6_Na^+^	203.0530	203.0515	0.9	0.35
5-(3-(dihydrocaffeoyl)oxy)- 2,3,4-trihydroxypentanoic acid	C_14_H_18_O_9_Na^+^	353.2643	353.2649	1.1	0.80
methyl 2,3,4-trihydroxy-5-((dihydroferuloyl)oxy) pentanoate	C_16_H_22_O_9_Na^+^	381.2974	381.2970	3.9	4.30
2-(hydroxymethyl)-6-(4(octyloxy)phenetoxy) tetrahydro-2H-pyran-3,4,5-triol	C_22_H_36_O_7_	413.2540	413.2669	2.7	1.17
Miquelianin	C_21_H_18_O_13_	479.0820	479.3503	3.3	0.83
Cholest-5-en-3-yl 3,4,5-trihydroxybenzoate	C_34_H_50_O_5_	561.3630	561.3635	1.2	0.11
Distearin	C_39_H_76_O_5_Na^+^	647.5530	647.5538	0.9	0.62
[(2R,3R)-3-(acetyloxymethyl)-2-(1,3-benzodioxol -5-ylmethyl)-4-(3,4,5-trimethoxyphenyl)butyl] hexadecanoate	C_40_H_60_O_9_	685.3900	685.4406	1.5	1.74
[(3S,8S,9S,10R,13R,14S,17R)-17-[(E,2R,5R)- 5-ethyl-6-methylhept-3-en-2-yl]-3-hydroxy-10,13- dimethyl-2,3,4,7,8,9,11,12,14,15,16,17-dodecahydro- 1H-cyclopenta[a]phenanthren-1-yl] (2E,4E,6E,8E,10E,12E)- docosa-2,4,6,8,10,12-hexaenoate	C_51_H_78_O_3_	739.6081	739.6101	1.2	1.13

^a^ Error [ppm]: Absolute value of the deviation between measured mass and theoretical mass of the selected peak in [ppm]. ^b^ Percentage of compound detected in the DI-ESI analysis. Results correspond to the products yield in triplicate.

## Data Availability

Data are contained within the article.
